# Supplementary material for: Spin Glass Behavior in Amorphous Cr2Ge2Te6 Phase‐Change Alloy
Source: Adv Sci (Weinh). 2023 Jun 6;10(23):2302444. doi: 10.1002/advs.202302444 (PMC10427411; doi:10.1002/advs.202302444)
Supplement: Supplementary file 1 — Supporting Information [file ADVS-10-2302444-s001.pdf]

ADVANCED SCIENCE
Open Access

Supporting Information

for *Adv. Sci.*, DOI 10.1002/advs.202302444

Spin Glass Behavior in Amorphous $Cr_2Ge_2Te_6$ Phase-Change Alloy

Xiaozhe Wang, Suyang Sun, Jiang-Jing Wang, Shuang Li, Jian Zhou, Oktay Aktas, Ming Xu, Volker L. Deringer, Riccardo Mazzarello, En Ma and Wei Zhang**

Supporting Information

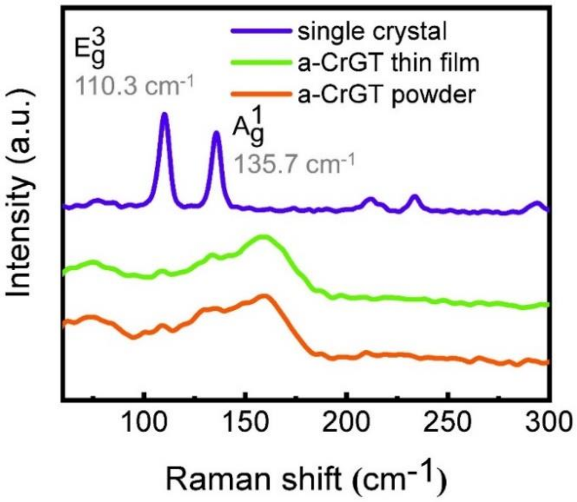

Figure S1. Raman spectroscopy experiments. In the CrGT single crystal, two primary modes are found at E_g^3 ~110.3 cm^{-1} and A_g^1 ~135.7 cm^{-1}. Two small humps are also visible in the two amorphous CrGT samples at these two frequencies. A primary peak is observed at ~158.6 cm^{-1} for both amorphous samples, and the two curves are overall very similar.

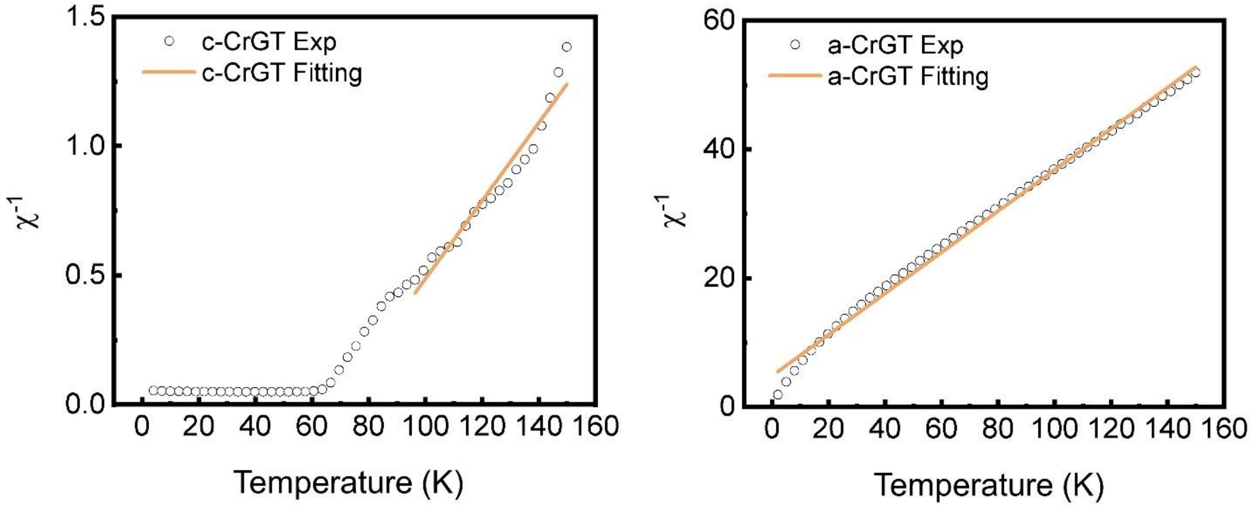

Figure S2. Magnetic analysis. The inverse susceptibility $\chi^{-1}(T)$ measured under 2000 Oe in ZFC. The curves measured for the single-crystal sample and amorphous powder sample are shown in the left and right panel, respectively, and are fitted by the Curie-Weiss law $\chi^{-1} = \frac{T-\theta}{c}$. Following Ref. [48], the single-crystal sample data above 100 K are used for linear fitting, and the obtained θ value equals 67.68 K, consistent with literature data. Regarding the amorphous sample, the data generally follow a linear change, and the obtained θ value equals –15.22 K.

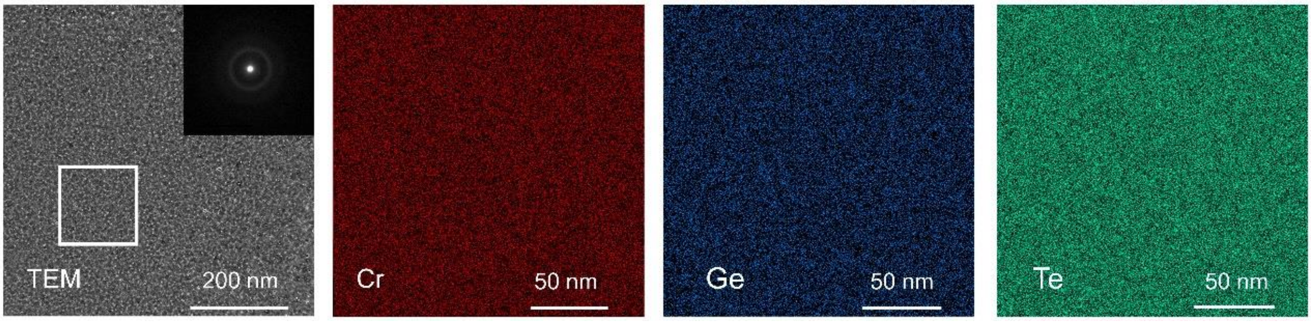

Figure S3. TEM characterization of as-deposited CrGT thin films. The bright-field TEM images and the corresponding SAED patterns with halo rings show the amorphous nature of the as-deposited CrGT films. The corresponding EDX mapping indicates that the distribution of Cr, Ge and Te atoms is homogenous at the length scale of tens of nanometers.

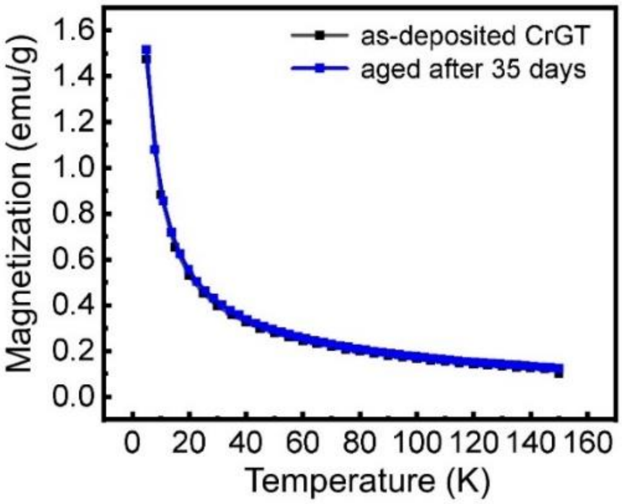

Figure S4. Magnetic measurements of a-CrGT before and after aging. The M-T curves measured for the a-CrGT powder sample before and after aging (after 35 days) are nearly identical. The applied magnetic field is 10 kOe.

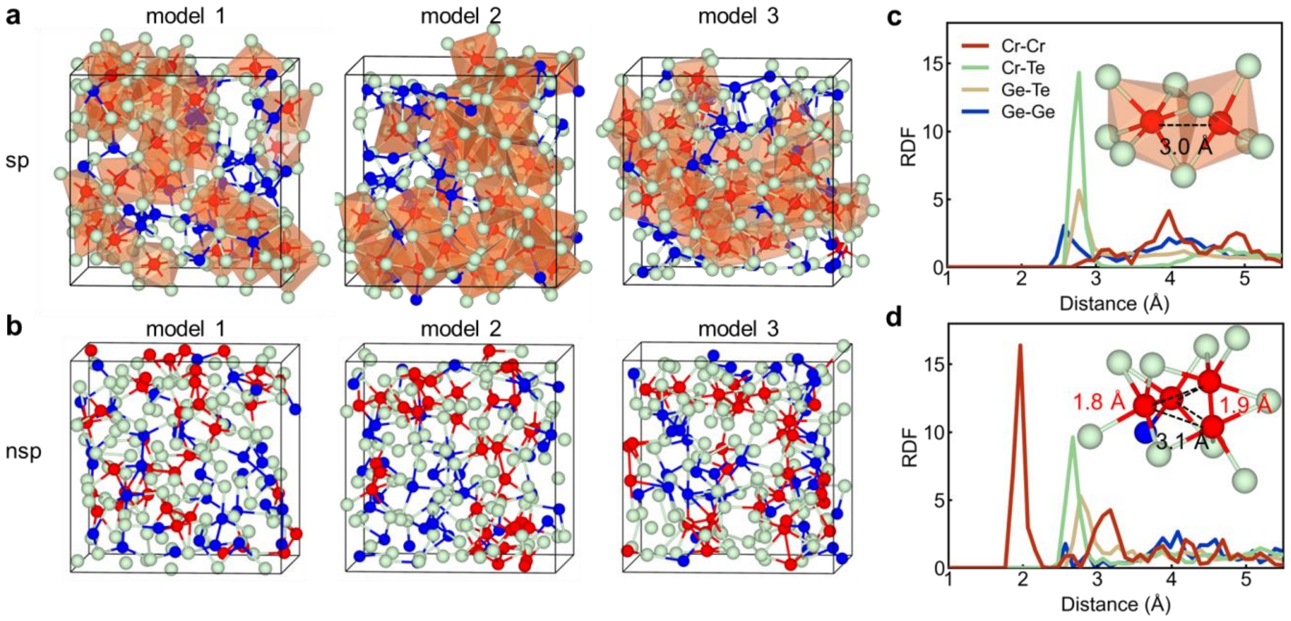

Figure S5. Snapshots and RDF curves of spin-polarized (sp) and non-spin-polarized (nsp) a-CrGT. a,b Snapshots of sp- (a) and nsp- (b) a-CrGT models. **c,d** The RDF curves of sp- (c) and nsp- (d) a-CrGT models annealing at 10K for 30ps with insets showing typical Cr local patterns. Cr, Ge and Te atoms are rendered as red, blue, and green spheres, respectively. Cr-centered octahedra are highlighted in orange. The sp-model 1 and the sp-RDF curves are shown in Fig. 5 in the main text.

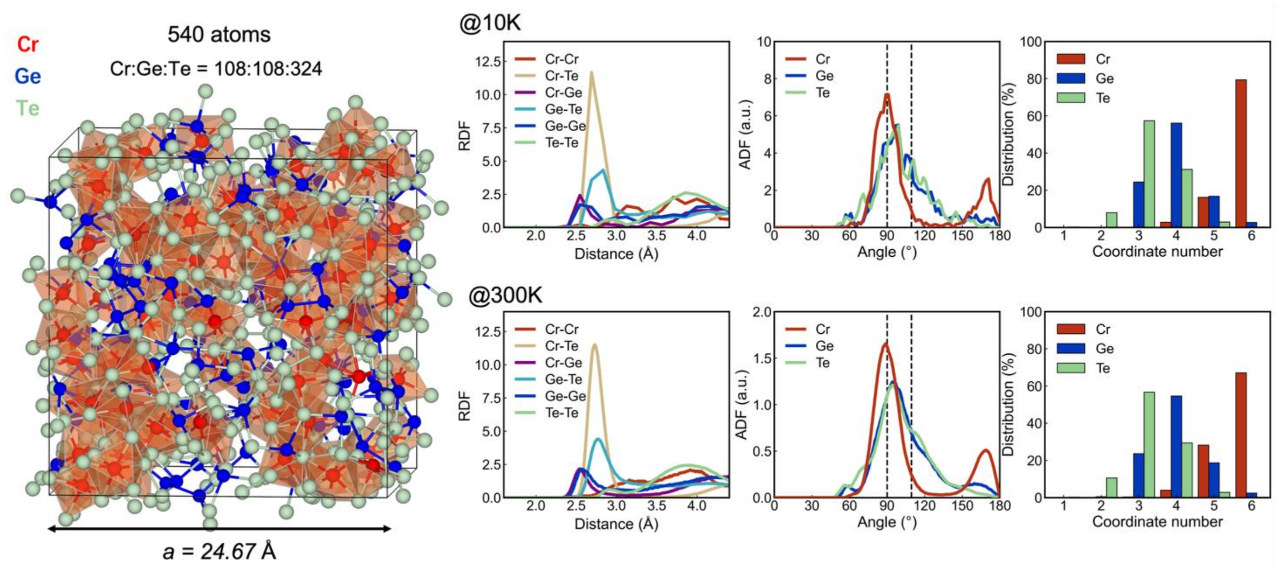

Figure S6. A large amorphous CrGT model of 540 atoms with spin polarization. The snapshot of the model together with RDF, ADF and CN distribution. The structural features of this big model are consistent with those of the small models shown in the main text.

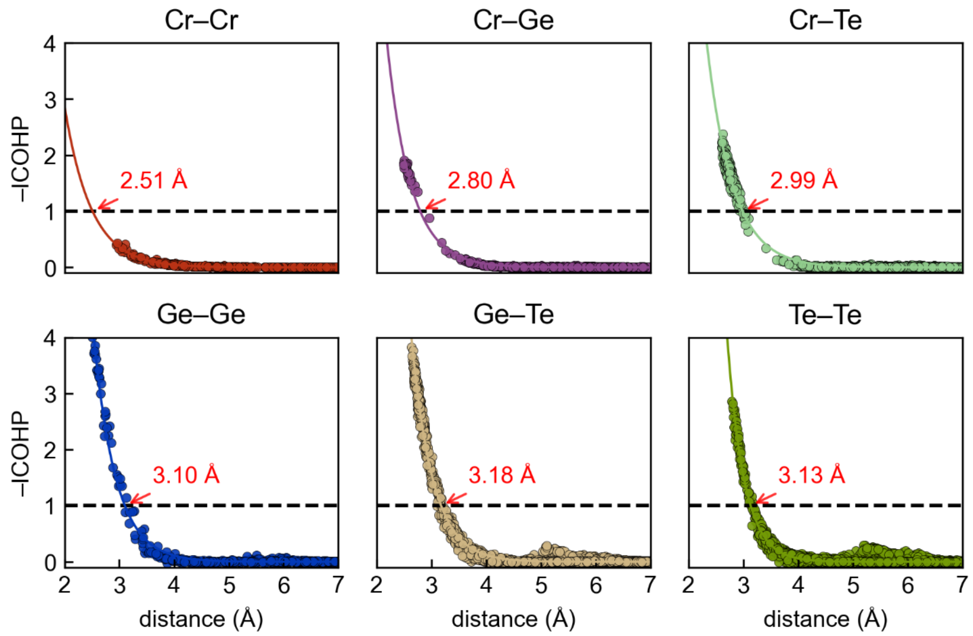

Figure S7. Cutoffs for interatomic distance determined using the −COHP integral. Following Ref. [58], the cutoff values for amorphous CrGT were determined as Cr−Cr 2.51 Å, Cr−Ge 2.80 Å, Cr−Te 2.99 Å, Ge−Ge 3.10 Å, Ge−Te 3.18 Å and Te−Te 3.13 Å.

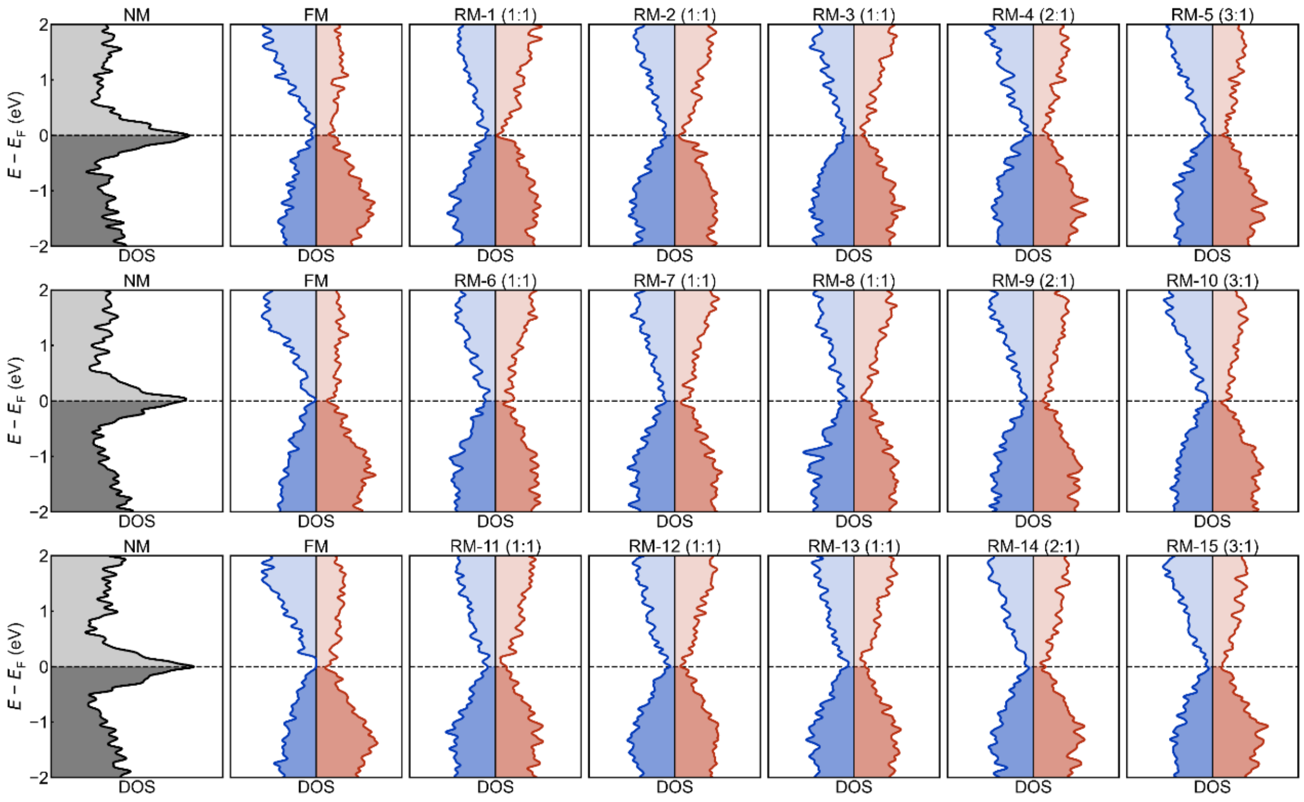

Figure S8. Electronic structure of spin-polarized a-CrGT models in NM, FM and RM configurations. For RM, finite magnetic moments were assigned to 1/2, 2/3 or 3/4 of the Cr atoms with positive values and the rest of them with negative values in a random fashion, denoted as (1:1), (2:1) and (3:1) in the corresponding panels. In total, 15 RM configurations were considered using 3

amorphous models.

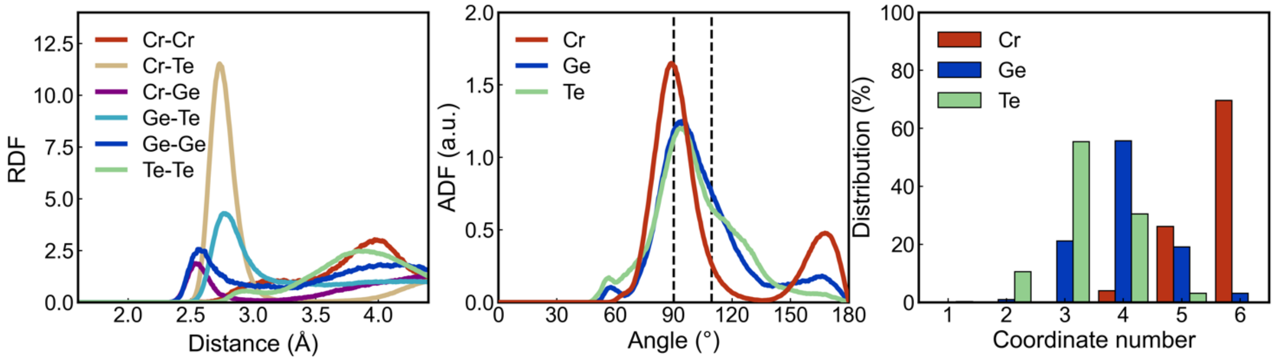

Figure S9. Structural analysis of spin-polarized a-CrGT models annealed at 300 K. The structural data were collected at 300K over 30 ps over three independent melt-quenched amorphous models.
